# Cerebellar ataxia and sensory ganglionopathy associated with light-chain myeloma

**DOI:** 10.1186/s40673-016-0060-4

**Published:** 2017-01-05

**Authors:** Panagiotis Zis, Dasappaiah Ganesh Rao, Bart E Wagner, Lucinda Nicholson-Goult, Nigel Hoggard, Marios Hadjivassiliou

**Affiliations:** 1 Academic Department of Neurosciences, Sheffield Teaching Hospitals NHS Foundation Trust, Sheffield, UK; 2 University of Sheffield, Royal Hallamshire Hospital, Royal Hallamshire Hospital, Glossop Rd, Sheffield, South Yorkshire S10 2JF UK; 3 Histopathology Department, Sheffield Teaching Hospitals NHS Foundation Trust, Sheffield, UK; 4 Department of Neuroradiology, Sheffield Teaching Hospitals NHS Foundaiton Trust, Sheffield, UK

**Keywords:** Sensory ganglionopathy, Ataxia, Cerebellar, Amyloid, Myeloma, Paraneoplastic

## Abstract

**Background:**

Cerebellar ataxia with sensory ganglionopathy is a rare neurological combination that can occur in some hereditary ataxias including mitochondrial diseases and in gluten sensitivity. Individually each condition can be a classic paraneoplastic neurological syndrome. We report a patient with this combination who was diagnosed with light-chain myeloma ten years after initial presentation.

**Case presentation:**

A 65-year-old Caucasian lady was referred to our Ataxia Clinic because of a 6-year history of progressive unsteadiness and a 2-year history of slurred speech. Past medical history included arterial hypertension. The patient was a non-smoker was not consuming alcohol excessively. There was no family history of ataxia.

Neurological examination revealed prominent gaze-evoked nystagmus, heel to shin ataxia, gait ataxia, reduced reflexes and loss of vibration sensation in the legs.

Cerebellar ataxia was confirmed using magnetic resonance spectroscopy of the cerebellum and sensory ganglionopathy using neurophysiological assessments including blink reflex study. A muscle biopsy that was arranged to explore the possibility of mitochondrial disease revealed amyloidosis. Urinalysis confirmed the presence of light chains. A bone marrow biopsy confirmed the diagnosis of light chain multiple myeloma.

**Conclusions:**

Whilst it could be argued that this could simply be a coincidence, the rarity of these conditions and the absence of an alternative aetiology for the neurological dysfunction argue in favour of a paraneoplastic phenomenon.

**Electronic supplementary material:**

The online version of this article (doi:10.1186/s40673-016-0060-4) contains supplementary material, which is available to authorized users.

## Background

Ataxia is a term used to describe poor control of movement, which manifests with unsteadiness and poor co-ordination. Ataxia can be mainly classified into cerebellar and sensory.

Cerebellar ataxia, resulting from a dysfunction of the cerebellum, can be inherited (e.g. Friedreich’s ataxia and spinocerenellar ataxias, known as SCAs) or acquired (such as immune mediated and paraneoplastic) [1].

Sensory ataxia results from a loss of proprioception and is usually caused by dysfunction within the sensory pathways, both peripheral and central. Sensory ataxia can therefore be seen in neuropathies that involve sensory fibers in isolation or in combination with motor fiber involvement. Sensory ataxia is more prominent in pure sensory neuropathies also called sensory neuronopathies [2]. Causes for sensory neuropathies or neuronopathies can be immune mediated [3] e.g. gluten sensitivity [4] Sjogren’s syndrome and paraneoplastic [5].

Cerebellar ataxia combined with sensory neuronopathy is a relatively rare neurological picture, which can be seen in some hereditary ataxias [2] (e.g. Friedreich’s ataxia and SCA18, mitochondrial disease) or as a result of exposure to toxins (e.g. amiodarone [6]).

We present a case with this combination with a rather unusual aetiology.

## Case presentation

A 65-year-old Caucasian lady was referred to our Ataxia Clinic because of a 6-year history of progressive unsteadiness and a 2-year history of slurred speech. Past medical history included arterial hypertension. The patient was a non-smoker was not consuming alcohol excessively. There was no family history of ataxia.

Neurological examination revealed prominent gaze-evoked nystagmus, heel to shin ataxia, gait ataxia, reduced reflexes and loss of vibration sensation in the legs.

Routine blood tests included full blood count, erythrocyte sedimentation rate, urea, creatinine, electrolytes, liver function tests, thyroid function tests, random glucose, Vitamin B12, folate, Vitamin E and folate, were all normal.

Extensive immunology screening included immunoglobulins and serum electrophoresis, antinuclear antibodies (ANA), anti-double stranded DNA (anti-dsDNA) antibodies, GAD antibodies, anti-neutrophil cytoplasmic antibodies (ANCA), thyroid peroxidase (TPO) antibodies, endomysium antibodies, transglutaminase antibodies and gliadin antibodies. From those, the IgA and IgM titer were low (IgA 0.63 g/L, normal values 0.8–4.0 and IgM 0.22 g/L, normal values 0.5–2.0), with no electrophoretic abnormality noted. C-ANCA antibodies were positive but PR3 antibodies were negative. Interestingly, the patient had the DQ8 human leukocyte antigen (HLA) serotype, which is commonly linked to autoimmune diseases, including coeliac disease [7].

**Fig. 1:**
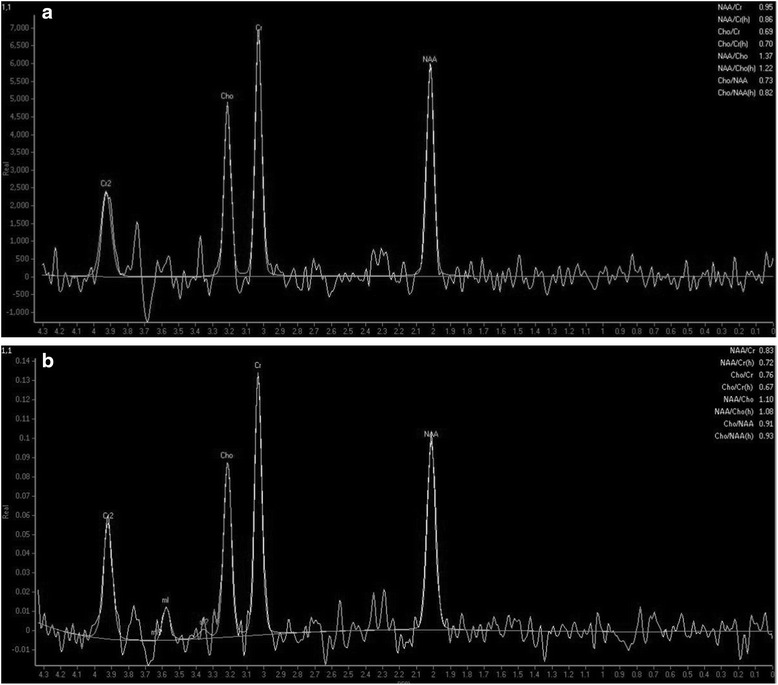
Spectral data obtained at intermediate echo-time by single-voxel 1H magnetic resonance spectroscopy of the cerebellar vermis. The amplitude of the resonances is measured on the Y-axis using an arbitrary scale. A low N-acetyl aspartate (NAA) to creatine (Cr) area ratio was found at first assessment with NAA/Cr 0.95, normal values >1.08 (A). MRS findings at 30 months show deterioration of NAA/Cr with a drop from 0.95 to 0.83 (B)

The MRI of the brain was unremarkable for the patient’s age, with no evidence of cerebellar atrophy in particular. However, the magnetic resonance spectroscopy (MRS) of the cerebellar vermis revealed a low N-acetyl aspartate (NAA) to Creatine (Cr) area ratio (NAA/Cr 0.95, normal values [8] > 1.0) (Fig. 1A). Low NAA/Cr ratio implies abnormal metabolic activity within the area of interest (voxel). It can be used as an indicator to cerebellar dysfunction [9].

Sensory conduction studies of the right median, right ulnar, right radial and both sural nerves showed attenuated amplitudes, when the motor conduction studies of the right median, right ulnar and right peroneal nerves were within normal limits. The electrically elicited blink reflex was tested bilaterally and revealed an asymmetric involvement of the trigeminal/facial nerve pathways, suggesting a diagnosis of sensory ganglionopathy [10].

As the combination of cerebellar ataxia with sensory ganglionopathy can be seen as part of hereditary ataxias, including mitochondrial diseases, the patient was initially tested and was negative for the common genetic ataxias (Fridriech’s ataxia, SCA1, SCA2, SCA3, SCA6 and SCA7). Subsequent further genetic testing using new generation sequencing was also negative and included the following genes; ABCB7, AFG3L2, APTX, ATM, ATP1A2, ATP1A3, ATP7B, C10orf2, CACNA1A, CACNB4, CYP2U1, CYP27A1, DDHD2, EEF2, FGF14, FTL, FXN, GBA, GBA2, IFRD1, ITPR1, KCNA1, KCNC3, KCND3, MTPAP, PDYN, PRKCG, PRRT2, SACS, SCN1A, SETX, SIL1, SLC16A2, SLC1A3, SLC2A1, SPG7, SPTBN2, TGM6, TTBK2, TTPA, VAMP1, ZFYVE26 (Additional file 1).

### Clinical course

**Fig. 2:**
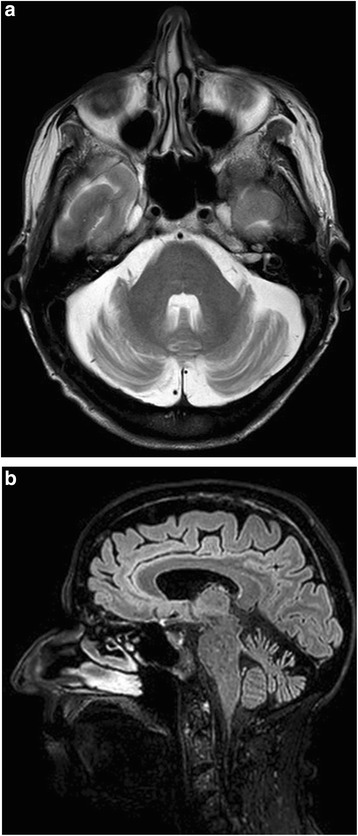
**a** Axial MRI T2 image showing atrophy of the cerebellar hemispheres (grade 1). **b** Sagittal MRI image, showing vermian grade 2 atrophy

The patient has been followed-up on a 6 monthly basis and remained relatively stable. Thirty months after the initial presentation to our clinic repeat nerve conduction studies and repeat MRI and MRS were arranged. The nerve conduction studies showed further deterioration of the sensory ganglionopathy. The MRI of the brain showed is mild to moderate atrophy of the vermis [11] (grade 2) and mild atrophy [11] (grade 1) of the hemispheres (Fig. 2), when the MRS showed further deterioration of the NAA/Cr ratios of the cerebellum (Fig. 1B).

**Fig. 3:**
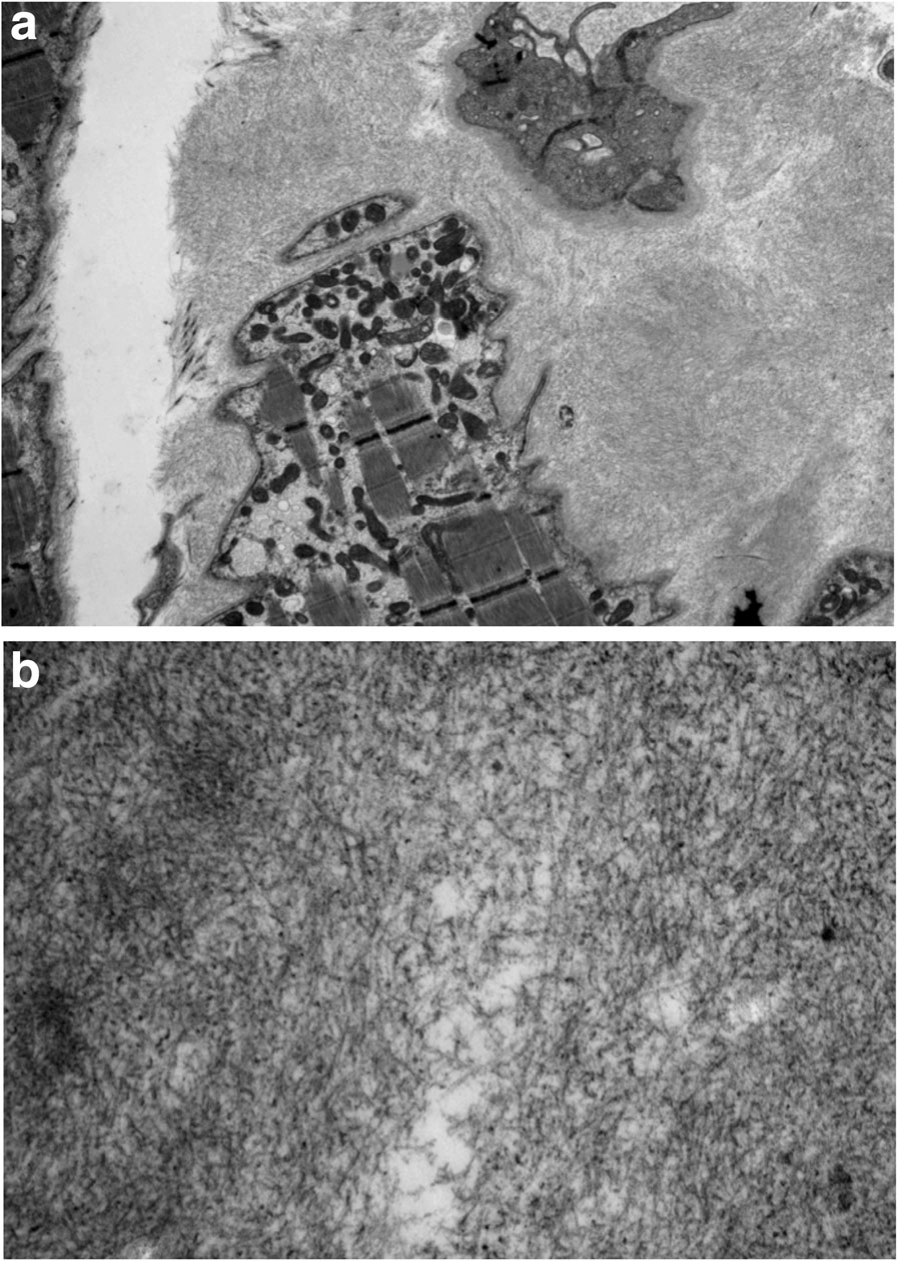
**a** Electron micrograph (original magnification × 2,600) of a mass of randomly arranged straight amyloid fibrils around a capillary (at the top of the image), and a skeletal muscle myocyte (at the bottom). **b** Electron micrograph (original magnification × 20,000) showing randomly arranged straight amyloid fibrils

In order to rule out mitochondrial disease the common mitochondrial mutations (MELAS, MERRF, NARP, POLG1) were tested and were negative and a muscle biopsy was arranged. Transmission electron microscopy of glutaraldehyde fixed muscle revealed the presence of endomysial amyloid. Histologically, no inflammation, necrosis, or inclusions were seen (Fig. 3). Urinalysis confirmed the presence of light chains. A bone marrow biopsy was performed confirming the diagnosis of light chain multiple myeloma. Scintigraphy showed predominantly cardiac involvement and the patient was referred for further treatment.

Table 1 summarizes the nerve conduction tests and the MRS findings across the clinical course of the patient.

**Table 1:** Neurophysiological and Magnetic Resonance Spectroscopy findings at baseline and follow-up visits

Neurological dysfunction in coeliac disease and	Neurological dysfunction in coeliac disease and	Neurological dysfunction in coeliac disease and	Neurological dysfunction in coeliac disease and	Neurological dysfunction in coeliac disease and	Neurological dysfunction in coeliac disease and	Neurological dysfunction in coeliac disease and	Neurological dysfunction in coeliac disease and	Neurological dysfunction in coeliac disease and	Neurological dysfunction in coeliac disease and	Neurological dysfunction in coeliac disease and	Neurological dysfunction in coeliac disease and	Neurological dysfunction in coeliac disease and	Neurological dysfunction in coeliac disease and
Baseline	11.7	8.8	6.3	X	0.8	0.8	2.9	0.7/0.7	10.6/16.3	60.5/55.3	60.7/55.5	0.95	1.04
F/U at 30 m	10.4	X	4.4	11.1	NR	0.5	NR	NR	X	X	X	0.83	1.17
F/U at 42 m	10.7	6.6	3.5	11.1	NR	NR	NR	NR	X	X	X	0.80	0.95
F/U at 54 m	X	X	X	X	X	X	X	X	X	X	X	0.79	0.80

*CMAP*; compound muscle action potential, *SNAP*; sensory nerve action potential, *L*; Left, *R*; Right, *NR*; no response, *X*; not done

Median and ulnar studies - orthodromic, radial, peroneal and sural - antidromic

cR2 = contralateral R2; iR2 = ipsilateral R2

## Discussion

Despite the extensive diagnostic work up for cerebellar ataxia combined with sensory ganglionopathy our patient was only diagnosed with light-chain multiple myeloma 10 years after the onset of the neurological symptoms. Until then, our working diagnosis based on extensive investigations was idiopathic sporadic cerebellar ataxia with sensory ganglionopathy, possibly auto-immune mediated.

Light-chain myeloma accounts for about 20% of multiple myeloma cases [12]. Three quarters of the light-chain myelomas are of kappa light chain and in only 25% the histological finding is the deposition of lambda light chain [12]. Therefore, the lambda light chain myeloma accounts for only the 5% of all multiple myeloma cases.

Neurological manifestations of light chain myeloma can be central or peripheral. Central manifestations most commonly result from cerebral or cerebellar plasmacytomas [13]. Occasionally patients may present with myelopathy secondary to paraspinal plasmacytomas [14]. Peripheral manifestations include radiculopathy secondary to fractures of the vertebrae, amyloid neuropathy and peripheral neuropathies related to a remote effect of the monoclonal gammopathy or to amyloidosis, in addition to the neurotoxicity of treatments, such as thalidomide and bortezomib [15].

Amyloid neuropathy occurs secondary to deposition of amyloid in the peripheral nerves. It usually leads to a symmetrical sensorimotor axonal neuropathy almost always with autonomic involvement. In our case, as implied by the nerve conduction studies, the pathology had to be confined to the dorsal root ganglia. In addition the patient had no autonomic dysfunction. It is therefore highly unlikely that the sensory ganglionopathy, that the patient had, was related to the amyloidosis.

Sub-acute sensory ganglionopathy as a paraneoplastic phenomenon is commonly associated with small cell lung cancer [16]. Other tumors associated with paranoplastic sensory neuronopathy include breast cancer, prostate cancer, ovarian cancer, bladder cancer, Hodgkin Lymphoma and sarcoma [17]. Cerebellar degeneration as a paraneoplastic phenomenon is associated with a broad spectrum of tumors including ovarian cancer, breast cancer, lung cancer and Hodgkin’s lymphoma [18]. To our knowledge, multiple myeloma has not been linked to any paraneoplastic neurological syndrome.

The recommended diagnostic criteria for paraneoplastic neurological syndromes [19] suggest that the cancer has to develop within five years of the diagnosis of neurological disorder. This time period has been based on reports showing that in the majority of cases the interval between the paraneoplastic neurological syndrome and the diagnosis of cancer is less than 5 years. In our case, the interval between the onset of the neurological symptoms and the diagnosis of the malignancy was ten years.

## Conclusions

To our knowledge this is the first case of cerebellar ataxia with sensory ganglionopathy associated with light chain myeloma. Whilst it could be argued that this could simply be a coincidence, the rarity of these conditions and the absence of an alternative aetiology for the neurological dysfunction argues in favour of a paraneoplastic phenomenon. As the myeloma in this case is not curable the chances of recovery with chemotherapy are remote. Indeed on the latest MR imaging there is significant progression of the spectroscopic abnormalities as well as the development of cerebellar atrophy.

## Additional file

Additional file 1: Ataxia panel performed. Diseases corresponding to the genes tested. (DOCX 104 kb)

## Abbreviations

ANA: Antinuclear antibodies
ANCA: Anti-neutrophil cytoplasmic antibodies
Cr: Creatine
dsDNA: Double stranded Deoxyribonucleic acid
GAD: Glutamic acid decarboxylase
MELAS: Mitochondrial encephalomyopathy, lactic acidosis, and stroke-like episodes
MERRF: Myoclonic Epilepsy with Ragged Red Fibers
MRI: Magnetic resonance imaging
MRS: Magnetic resonance spectroscopy
NAA: N-acetyl aspartate (NAA)
NARP: Neuropathy, ataxia, and retinitis pigmentosa
SCA: Spinocerebellar ataxia
TPO: Thyroid peroxidase
