# Supplementary material for: Cerebellar ataxia and sensory ganglionopathy associated with light-chain myeloma
Source: Cerebellum Ataxias. 2017 Jan 5;4:1. doi: 10.1186/s40673-016-0060-4 (PMC5217606; doi:10.1186/s40673-016-0060-4)
Supplement: Additional file 1: — Ataxia panel performed. Diseases corresponding to the genes tested. (DOCX 104 kb) [file 40673_2016_60_MOESM1_ESM.docx]

| ABCB7 | X-linked sideroblastic anemia with ataxia |
| --- | --- |
| AFG3L2 | Spastic ataxia 5, autosomal recessive  SCA28 |
| APTX | Ataxia with oculomotor apraxia |
| ATM | Ataxia-telangiectasia |
| ATP1A2 | Episodic ataxia, hemiplegia, and seizures. |
| ATP1A3 | CAPOS syndrome |
| ATP7B | Wilson’s disease |
| C10orf2 | Ataxia neuropathy spectrum |
| CACNA1A | Episodic ataxia type 2  SCA6 |
| CACNB4 | Episodic ataxia type 5 |
| CYP2U1 | SPG56 |
| CYP27A1 | Cerebrotendinous Xanthomatosis |
| DDHD2 | SPG564 |
| EEF2 | SCA26 |
| FGF14 | SCA27 |
| FTL | Neuroferritinopathy |
| FXN | Friedreich’s ataxia |
| GBA | SCA3 |
| GBA2 | Autosomal-recessive cerebellar ataxia |
| IFRD1 | SCA18 |
| ITPR1 | SCA15  SCA29 |
| KCNA1 | Episodic ataxia type 1 |
| KCNC3 | SCA13 |
| KCND3 | SCA22 |
| MTPAP | Spastic ataxia 4, autosomal recessive |
| PDYN | SCA23 |
| PRKCG | SCA14 |
| PRRT2 | Paroxysmal Kinesigenic Dyskinesia and Episodic ataxia |
| SACS | Autosomal recessive spastic ataxia of Charlevoix-Saguenay |
| SCN1A | Severe myoclonic epilepsy of infancy |
| SETX | Ataxia with oculomotor apraxia |
| SIL1 | Marinesco-Sjögren syndrome |
| SLC16A2 | Allan-Herndon-Dudley Syndrome |
| SLC1A3 | Episodic ataxia type 6 |
| SLC2A1 | Exercise-induced Paroxysmal Dyskinesia and Episodic ataxia |
| SPG7 | Hereditary spastic paraplegia 7 |
| SPTBN2 | SCA5 |
| TGM6 | SCA35 |
| TTBK2 | SCA11 |
| TTPA | Ataxia with hereditary vitamin E deficiency |
| VAMP1 | Spastic ataxia 1 |
| ZFYVE26 | Hereditary spastic paraplegia 15 |
|  |  |
